# The anterior insular cortex processes social recognition memory

**DOI:** 10.1038/s41598-023-38044-6

**Published:** 2023-07-05

**Authors:** Ji-You Min, Sanggeon Park, Jeiwon Cho, Yeowool Huh

**Affiliations:** 1 Department of Brain and Cognitive Sciences, Scranton College, Ewha Womans University, Seoul, 03760 Republic of Korea; 2 Brain Disease Research Institute, Ewha Brain Institute, Ewha Womans University, Seoul, 03760 Republic of Korea; 3 Department of Medical Science, College of Medicine, Catholic Kwandong University, Gangneung‑si, 25601 Republic of Korea; 4 Translational Brain Research Center, International St. Mary’s Hospital, Catholic Kwandong University, Incheon, 22711 Republic of Korea

**Keywords:** Neuroscience, Cognitive neuroscience, Social behaviour

## Abstract

Impaired social abilities are characteristics of a variety of psychiatric disorders such as schizophrenia, autism spectrum disorder, and bipolar disorder. Studies consistently implicated the relationship between the anterior insular cortex (aIC) and social ability, however, how the aIC involves in processing specific subtypes of social ability was uninvestigated. We, therefore, investigated whether the absence or presence of the aIC affects the social behaviors of mice. We found that electrolytic lesions of the aIC specifically impaired mice’s ability to recognize a novel stranger mouse, while the sociability of the aIC-lesioned mice was intact. Interestingly, the aIC-lesioned mice were still distinguished between a mouse that had been housed together before the aIC lesion and a novel mouse, supporting that retrieval of social recognition memory may not involve the aIC. Additional behavioral tests revealed that this specific social ability impairment induced by the aIC lesion was not due to impairment in olfaction, learning and memory, locomotion, or anxiety levels. Together our data suggest that the aIC is specifically involved in processing social recognition memory, but not necessarily involved in retrieving it.

## Introduction

Social skills are crucial for social interactions. Disruption in the social brain network is thought to underlie impaired social abilities of many psychiatric disorders including schizophrenia, autism spectrum disorder (ASD), and bipolar disorder^1–3^. Social abilities require a complex interplay of perceptual, motor, emotional, and social context-sensitive decision-making processes. Due to its multifaceted nature, social skills are processed by various brain regions and some of the well-known regions within the social brain networks include the amygdala, medial prefrontal cortex (mPFC), nucleus accumbens (NAc), hippocampus, and hypothalamus^4^. Investigating a brain region that is less intensively studied in relation to social skills will provide additional insights into understanding its complexity and possibly finding new treatments for patients with social ability deficits.

The role of the insular cortex (IC) in social skills is less well-studied. Clinical studies, however, consistently reported the relationship between IC and social abilities. ASD patients displayed IC hypo-activation only during social tasks^5^. Reduced connectivity between the amygdala and IC during social judgment was found in patients with schizophrenia^6^. Insular hyperactivity to social stimuli was reported in people with Tourette syndrome^7^. Patients with prosopagnosia, also known as face blindness, often had lesions that included the IC^8–10^. Also, the activity of the left IC was reported to be associated with the social judgment of facial emotions^11^.

The subregions of the IC have distinct connectivity patterns. The anterior insular cortex (aIC) has stronger connections with the high-order cortical and subcortical brain regions while the posterior insular cortex (pIC) has connections with brain regions processing sensory information^12^. Notably, the aIC has connections with the brain regions within the well-known social network such as the mPFC, NAc, and amygdala. In support of the role of the aIC in sociality, a human study reports that people with a higher social network index, a composite measure of an individual’s network diversity, size, and complexity, had greater volume and sulcus depth of the aIC^13^. Animal studies also suggest the involvement of the aIC in social behaviors: chemogenetically activating the aIC restored rescue behaviors in heroin administered rats^14^ whereas chemogenetically inhibiting the aIC reduced targeted helping behavior in rats^15^. The pIC, which has relatively less connection with the social brain regions than the aIC, was also found to have roles in social behaviors; recent animal studies demonstrated that the pIC regulates behavior to approach or avoid in response to social stimuli in rats^16^ and activating the pIC → CeA acutely interrupted ongoing social interactions in mice^17^.

Different connectivity patterns of the aIC and pIC are expected to confer them with differential roles, but the role of the aIC in processing specific type of social ability remain relatively uninvestigated. We, therefore, investigated the role of the aIC in different types of social abilities using electrolytic lesion and various behavioral measurements in male mice. Sex differences have been reported to affect social recognition in both humans and animals^18^. To reduce variabilities, this study only investigated male mice. We found that lesion of the aIC specifically impaired social recognition memory, but other abilities such as sociability with other mice, odor discrimination, and fear memory formation and extinction were spared. The aIC lesioned mice still recognized cage-mates, suggesting that retrieval of social recognition memory is not mediated by the aIC.

## Results

### Lesion of the aIC impairs social recognition memory

**Figure 1:**
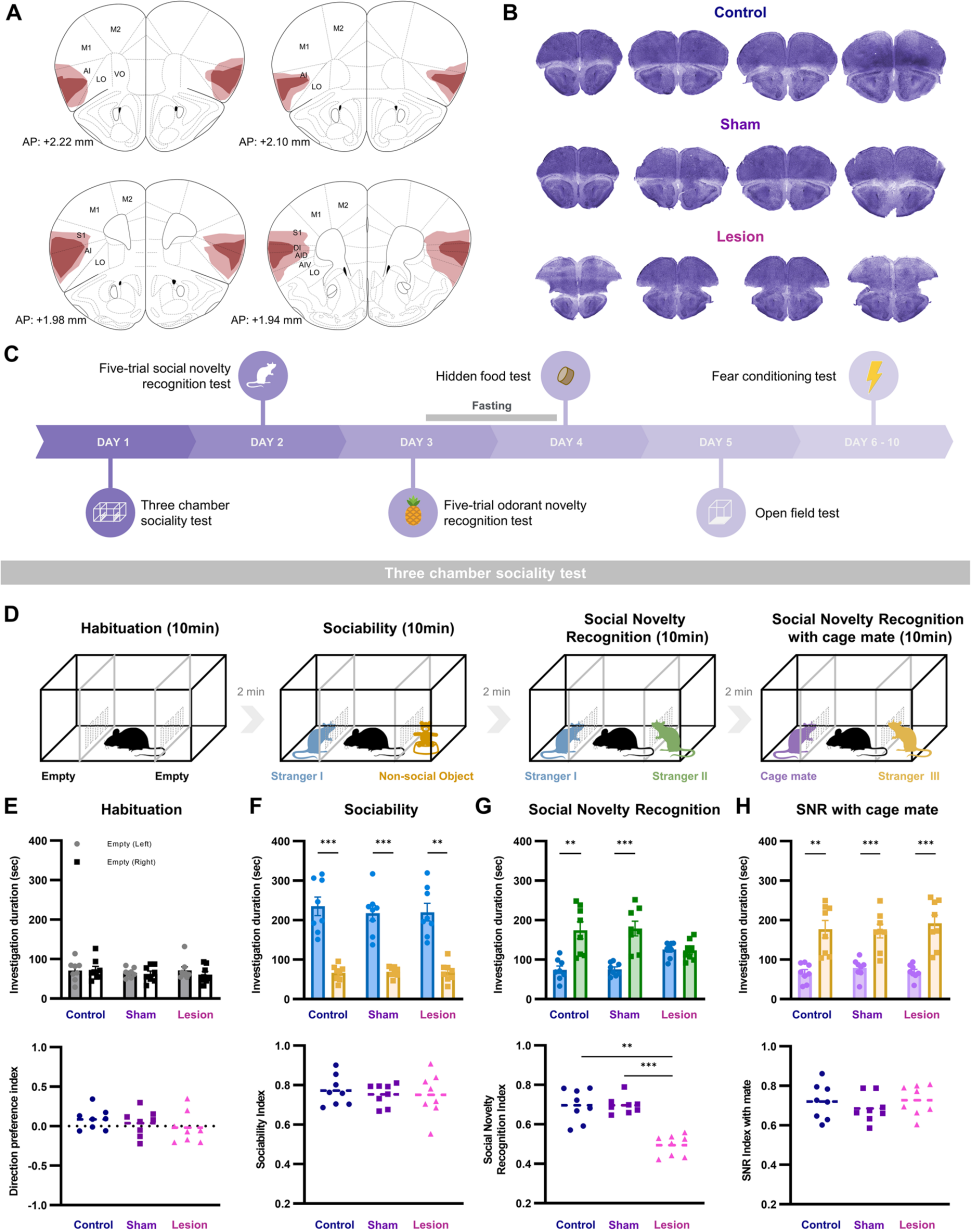
The aIC-lesioned mice showed specific deficits in forming social novelty recognition memory with other social abilities preserved, (**A**) Schematic drawings of the average lesion locations of mice in the lesion group. Darker red areas indicate complete ablation; lighter red areas indicate partial tissue damage. (**B**) Representative pictures of cresyl-violet stained brain slices from control, sham, and aIC-lesion groups. (**C**) Timeline of the behavioral tests. (**D**) Sequence of the behavioral experiment paradigms of the linear chamber test. (**E**) Mean investigation duration of the three groups during the habituation session (upper) and the direction preference index of the three groups (lower). (**F**) Mean investigation duration of each group toward a social stimulus and a non-social object (upper) and the sociability index of the three groups (lower). (**G**) Social novelty recognition test (SNR) with new stranger mouse (stranger II) introduced. Mean investigation duration of each group during the test (upper) and the social novelty recognition index of the three groups (lower). (**H**) Social novelty recognition test with a cage mate and stranger III. Mean investigation duration of each group during the test (upper) and the social novelty recognition index of the three groups (lower). (**E**–**H**) All data are presented as mean ± standard error of mean (SEM). Paired t-test was used to compare within subject differences (left chamber vs. right chamber, stranger I versus non-social object, stranger I vs. stranger II, and cage mate vs. stranger III). One-way ANOVA was used to compare difference between different groups. * p < 0.05, ** p < 0.01, *** p < 0.001.

To assess the role of the aIC in social recognition, behaviors of aIC-lesioned mice (lesion; *n* = 8), sham surgery mice (sham; *n* = 8), and no surgery control mice (control; *n* = 8) were compared in three-chamber tests (see methods for detail). The lesion group received bilateral electrolytic lesions of the aIC, while the sham group underwent the same surgical procedure without the electrolytic lesion. The control group underwent identical procedures without any surgery. Complete lesions were mostly restricted within the aIC, but in some cases, cell death partially infringed into the primary somatosensory cortex. All aIC-lesion subjects had approximately equal aIC lesion sizes in both hemispheres (Supplementary Fig. S1). Schematic drawings of the average lesion locations of mice in the lesion group (Fig. 1A) and histological samples of the three groups are shown in Fig. 1B.

The timeline of all the behavioral tests carried out are outlined in Fig. 1C. Sequence of the three chamber sociality test is drawn in Fig. 1D. First, behaviors of mice were monitored when both chambers were empty. None of the groups showed preference towards the left or the right chamber, measured with investigation duration (Fig. 1E, upper), and there were no difference in preference between groups (Fig. 1E, lower). During the sociability test, which tests the propensity of mice to prefer a conspecific over a non-social object, mice in all groups showed a strong preference towards the stranger I (paired t-test; lesion *p* = 0.0013, sham *p* = 0.00009, control *p* = 0.00032; Fig. 1F, upper). The aIC lesioned mice still preferred to interact with a conspecific than an object. Sociability index confirms that sociability of mice, measure with social preference, is unimpaired by the aIC lesion (Fig. 1F, lower).

During the social novelty recognition test which compares behaviors towards an earlier-exposed stranger (stranger I) and a novel stranger (stranger II) mice, the sham and control mice preferred the novel over the earlier-exposed stranger (paired t-test; sham *p* = 0.00028, control *p* = 0.0016; Fig. 1G, upper). The lesion group, however, did not distinguish between stranger I and stranger II, suggesting that the ability to recognize the previously exposed social stimulus is impaired with aIC lesion. Compared to the sham and control, the ability of the aIC lesioned mice to recognize social novelty was significantly impaired (one-way ANOVA with post hoc Tukey’s test, F(2,21) = 14.961; *p* = 0.000786; lesion vs. sham *p* = 0.0009; lesion vs. control *p* = 0.003; Fig. 1G, lower).

Although social novelty recognition was impaired by lesion in the aIC, lesioned mice were still able to distinguish between a cage mate that had been housed together before the lesion was made. The lesion group showed significant preference towards a novel stranger (stranger III) over their cage mates just like the sham and control groups (paired t-test; lesion *p* = 0.0006, sham *p* = 0.0004, control *p* = 0.0012; Fig. 1H, upper) and social novelty recognition index of the three groups were similar (Fig. 1H, lower). This suggest that the aIC may be involved in the formation of social recognition memory, but not necessarily involved in the retrieval of already formed social recognition memory.

## Social recognition memory specific impairment by aIC lesion

**Figure 2:**
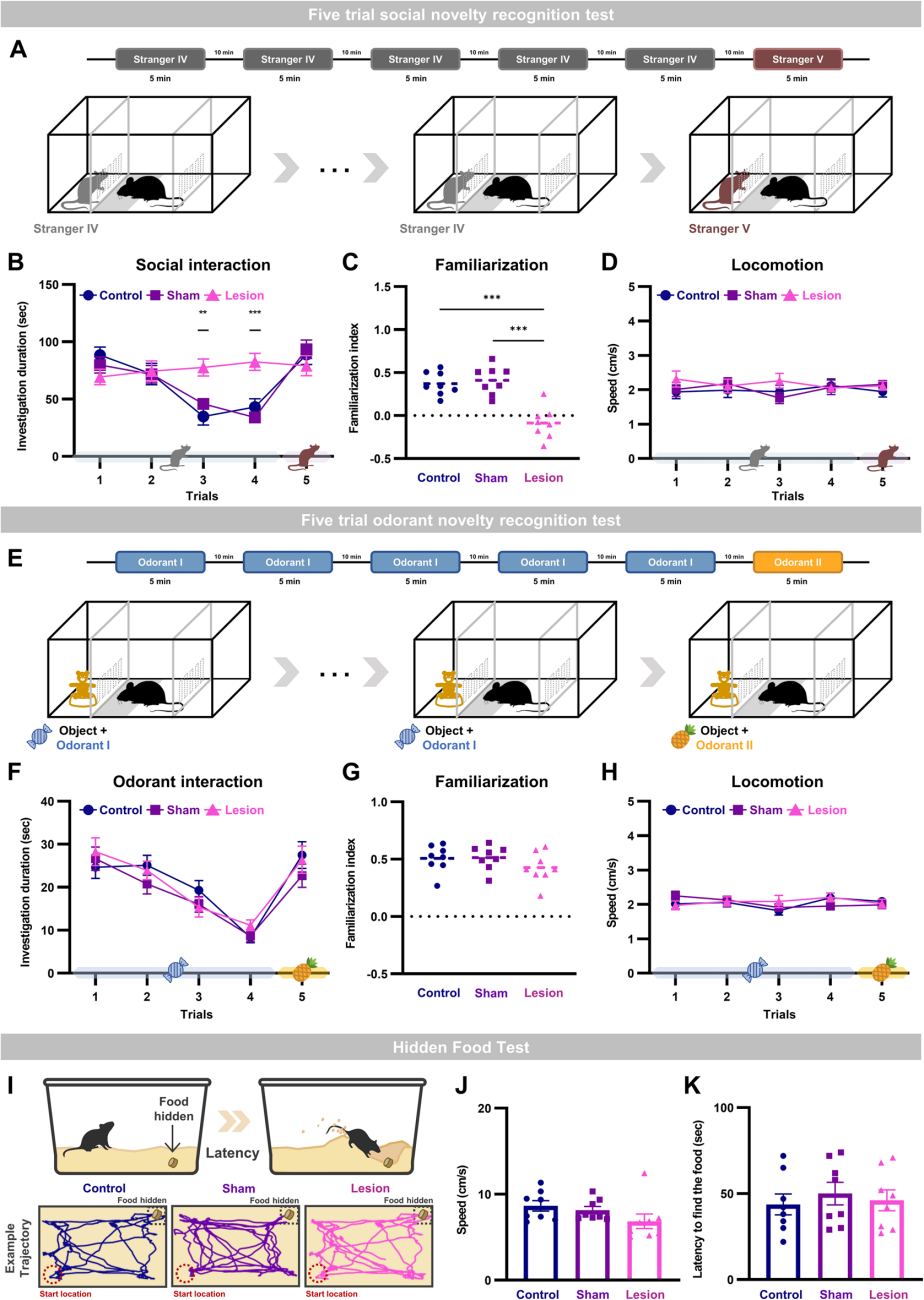
Social recognition memory specific impairment with aIC lesion demonstrated with the five-trial social novelty recognition test. (**A**) Experimental scheme of the 5-trial social novelty recognition test. (**B**) Investigation duration of the five-trial social novelty recognition test (one-way ANOVA between groups for each trial, **P < 0.01, ***P < 0.001). (**C**) Familiarization index of the three groups (see methods for detail) for the five-trial social novelty recognition test (one-way ANOVA with Tukey’s post-hoc test; ***P < 0.001). (**D**) Mean movement speeds of the three groups for the five-trial social novelty recognition test. (**E**) Experimental scheme of the 5-trial odorant novelty recognition test. (**F**) Investigation duration during of the five-trial odorant novelty recognition test. (**G**) Familiarization index of the three groups for the five-trials odorant novelty recognition test. (**H**) Mean movement speeds of the three groups for the five-trial odorant novelty recognition test. (**I**) Schematic drawings of the hidden food test (upper). Representative trace plots from one mouse in each group during the hidden food test (lower). (**J**) Mean movement speed of the three groups. (**K**) Mean latency to find food of the three groups.

To further analyze whether short-term social recognition memory may be impaired by lesion in the aIC, we used the five-trial social novelty recognition test (Fig. 2A). In the experiment, behaviors of mice towards an empty chamber and the same mouse (stranger IV) were monitored for four consecutive trials, then a novel mouse (stranger V) was introduced in the fifth trial. If a mouse remembers previous encounters, forming short-term social recognition memory, then social interactions will progressively decrease with consecutive encounters.

As expected, sham and control groups progressively decreased the investigation duration towards the stranger IV during the four trials (two-way repeated measures ANOVA with Bonferroni post-hoc test, F_group_(2,21) = 2.564, *p* = 0.101; F_trials_(3,63) = 29.305, *p* = 5.5353E-12; F_group*trials_(6,63) = 15.163, *p* = 1.1796E-10), but increased interaction time when a novel mouse was introduced in the fifth trial (Fig. 2B). The lesion group, in contrast, maintained the investigation duration throughout the five trials (Fig. 2B). The social familiarization index confirms that aIC lesion impaired mice’s ability to get familiar with mice that it socially encountered repeatedly (one-way ANOVA with post hoc Tukey’s test; F(2,21) = 22.984, *p* = 0.0000051; lesion vs. control *p* = 0.000042; lesion vs. sham *p* = 0.000013; Fig. 2C). The difference in social recognition behavior between groups is not due to difference in locomotion, since movement speed was constant throughout all trials and there was no difference between groups (Fig. 2D).

We then investigated whether this impairment in social recognition induced by aIC lesion was due to impaired olfactory system. For the experiment, the five-trial odorant novelty recognition test with a non-social object and two distinct odorants (bubble gum and pineapple scent) was used (Fig. 2E). Interestingly, there were no behavioral differences between groups (Fig. 2F). All three groups progressively decreased the investigation duration during the first four trials with the same odorant (two-way repeated measures ANOVA with Bonferroni post-hoc test; F_group_(2,21) = 0.201, *p* = 0.819; F_trials_(3,63) = 52.767, *p* = 7.3204E-26; F_group*trials_(6,63) = 2.808, *p* = 0.017428). When a new odorant was introduced in the fifth trial the investigation duration was reinstated and familiarization index was similar in all three groups (Fig. 2G). Locomotion during the odorant test was also consistent throughout trials and similar between groups (Fig. 2H). A hidden food test which tests the ability of mice to sniff and find food buried underneath bedding material (Fig. 2I) also confirmed that olfaction was intact by the aIC lesion. Neither locomotion nor latency to find a hidden food pellet was different between groups (Fig. 2J and K). The results suggest that the ability of aIC lesioned mice to recognize novel odorant and to form short-term odor recognition memory is unaffected.

## Locomotion and fear learning and memory intact in aIC lesioned mice

**Figure 3:**
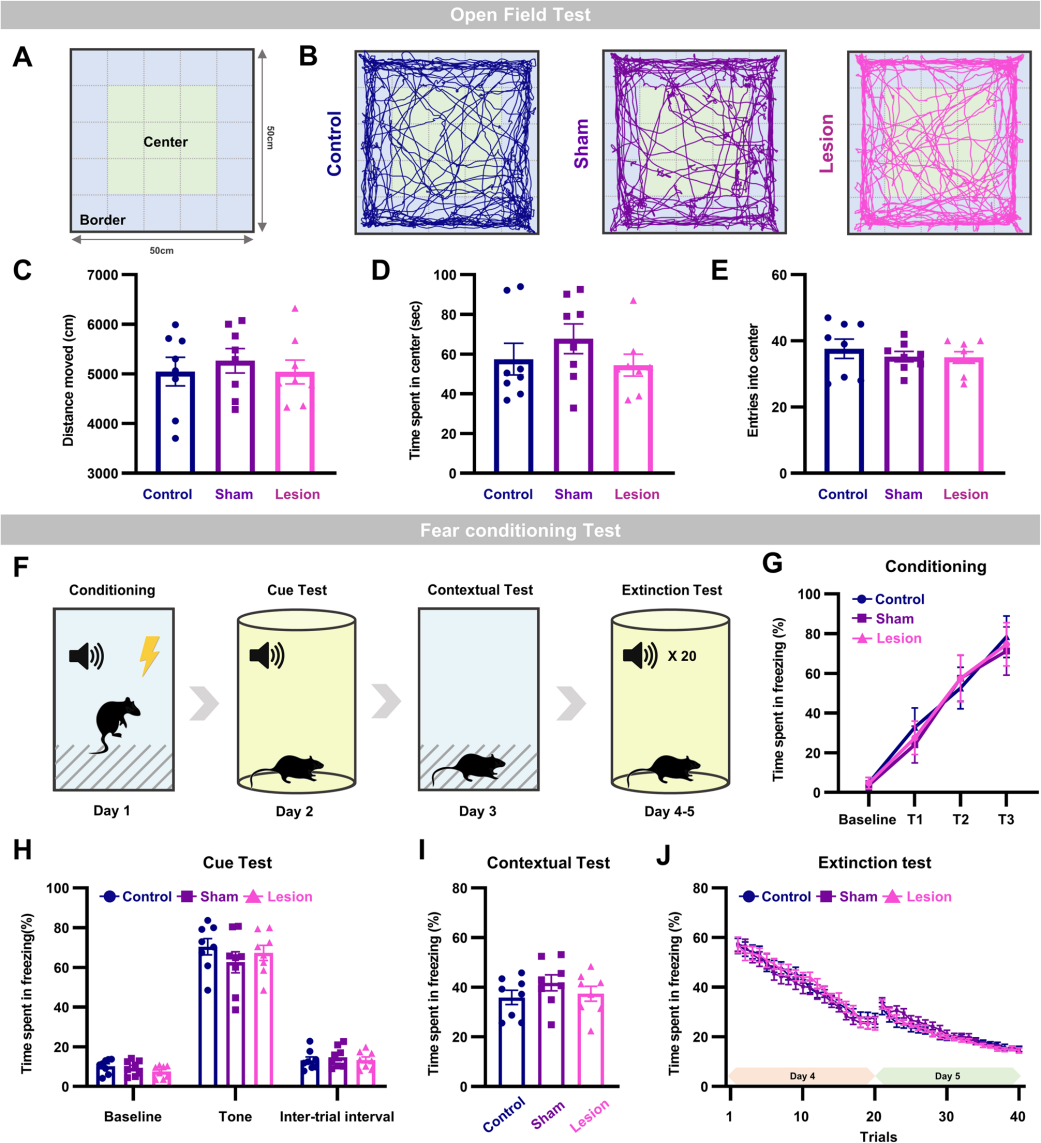
Locomotion, and fear learning and memory preserved in mice with aIC lesion, (**A**) Schematic drawings of the open field test. Within 10 cm from the wall was considered a border while the rest was considered central. (**B**) Representative trajectory of each group during the open field test. (**C**) Mean distance traveled of the three groups. (**D**) Mean time spent in the center of the three groups. (**E**) Mean entries into the center of the three groups. (**F**) Experimental scheme of fear conditioning and extinction protocol. (**G**) Fear memory acquisition of the three groups. (**H**) Mean freezing duration during the auditory cued fear expression test. (**I**) Mean freezing duration during the contextual fear test. (**J**) Fear extinction test of the three groups. (**C**–**E** and **G**–**J**) All data are presented as mean ± SEM. One-way ANOVA with Tukey’s post-hoc test was used to compare means between groups.

We investigated whether locomotion or other types of learning and memory is disrupted by lesion in the aIC with the open field test and fear learning and extinction tests. The open field test analysis parameters and sample trajectory of each group are shown in Fig, 3A and B. Consistent with other locomotion tests, the open field test showed that there was no difference in the total distance travelled between groups (Fig. 3C). The anxiety level, measured with the time spent in the center and number of entries into the center, were also similar between groups (Fig. 3D and E). This result supports that neither locomotion nor anxiety level influenced the observed social recognition memory deficit in aIC lesioned mice.

Protocols used for the classical fear condition and fear extinction tests is drawn in Fig. 3F. Mice were conditioned to three trials of tone (CS) that co-terminated with an electrical foot shock (US). Acquisition of fear during the three trials of conditioning were indistinguishable between groups (Fig. 3G). Mice in all three groups acquired tone-associated (CS: cued) fear memory tested the next day (Fig. 3H). Similarly, contextual fear memory tested on the third day was also intact in all three groups (Fig. 3I). The ability of aIC lesioned mice to learn fear extinction was also preserved and did not differ with the sham and control groups (Fig. 3J). Acquisition, storage, and retrieval of cued and contextual fear memory, and learning of fear extinction were unaffected even in the absence of aIC. Series of experiments collectively suggest that lesion of the aIC induces specific impairment in social recognition memory that is not due to impairment in olfaction or other learning and memory processes.

## Discussion

This study reveals the specific role of the aIC in processing social recognition memory. Interestingly, the deficit induced by aIC lesion was restricted to social recognition memory formation, whereas the propensity to spend more time with conspecifics (socialize) over a non-social object or the ability to recognize a cage mate (retrieval of social recognition memory) was unaffected. Also, olfactory discrimination memory and fear memory were unaffected by the aIC lesion, stressing the precise role of the aIC in processing social recognition memory.

Social abilities are complex and therefore expected to be processed by coordinated activity of multiple brain areas. One brain region may play a more dominant role in processing certain types of social ability, since cytotoxic lesions of the hippocampus increased social investigation, but did not impair social-recognition memory^19^. Clinical studies report that different social abilities are impaired in different cases. Patients with social anxiety disorder had a significant deficiency in social interaction with others, but their social novelty recognition was intact^20^. Also, clincial studies report that patients with semantic dementia have distinct impairments in different subtypes of social abilities, depending on the regions of significant brain volume loss^9, 21^. In line with these findings, our study suggests that the aIC plays a critical role in forming social recognition memory, since the ability to socialize with a conspecific and recognize a cage mate was spared even with lesions in the aIC.

Regarding specialized roles that different brain areas play, the aIC and pIC are expected to have specialized functions in social abilities. The pIC has connections with the sensory and limbic brain regions and, therefore, suggested to integrate multimodal sensory and limbic information^12^. In support, animal studies revealed that the pIC is involved in emotion-related social behavior, such as approaching or avoiding a stressed conspecific^16^. Modulating the projection of the pIC to emotion regulating amygdala also changed social behavior since activating the pIC → CeA acutely interrupted ongoing social interactions in mice^22^. The aIC, in contrast, has more connection with higher-order brain structures such as the mPFC and medial thalamus. How the different connectivity patterns confer different roles is elusive, but several studies have implicated the involvement of the aIC in social abilities. Human studies report a positive correlation between volume and sulcal depth of the aIC and social network index, a composite measure of an individual’s network diversity, size, and complexity^13^. Increased activity of the right aIC was positively correlated with the level of social anxiety^23^. A recent animal study report that the aIC is necessary for social novelty recognition^24^, consistent with our study.

Our study suggests that aIC lesion may induce deficit in the acquisition or consolidation of social recognition memory. In the three-chamber sociability test, aIC-lesioned mice had no preference in investigating a mouse that they had encountered before (stranger I) over a new mouse (stranger II) (Fig. 2G). The aIC-lesioned mice still preferred to investigate a new mouse (stranger III) over a cage-mate (Fig. 2G), suggesting that the ability to learn or consolidate social recognition memory may be impaired by the absence of the aIC. In support, the aIC-lesioned mice maintained their investigation duration towards a mouse (stranger IV) that was presented repeatedly over four trials (Fig. 2B). Interestingly, other types of learning and memories such as odor recognition memory and fear learning and memory were intact. Consistent with our findings, other studies report that odor detection or recognition may not involve the IC even though the IC is known to process olfaction^25^. Other studies also show that, unlike the pIC, the aIC does not involve in the acquisition of spatial memory (tested with water-maze) or conditioned taste aversion^26^, suggesting that the aIC could be more specialized in learning or consolidating social memories.

Our study's open field test and sociability results suggest that absence of the aIC neither affects the general anxiety nor the social anxiety levels. A human study, interestingly, reports a positive correlation between social anxiety level and aIC activity^23^. This may be related to the suggested role of the aIC in monitoring one’s internal state and self-awareness^27^, as being more self-aware may increase social nervousness. The results from our study and the human study together suggest that the activity of the aIC may be important in mediating social anxiety and that the absence of the aIC does not necessarily affect the level of social anxiety. Although this study did not investigate the relationship between the aIC and social anxiety, the results imply a possible link, and it would be interesting to figure out the detailed mechanism of aIC mediating social anxiety in future studies.

Although our study showed that aIC lesions impair social recognition memory, this may be specific to males since  another study reports that sex hormones and gender differentially affect social recognition^18^. Differences in social interest and oxytocin receptors have also been reported in rats^28^. In the study, male rats showed significantly higher oxytocin receptor binding density compared to females in several forebrain regions including the anterior insular cortex. Likewise, the aIC may have a different role in females and it remains to be determined in future studies.

Findings of our study highlight the role of the aIC in processing a specific type of social ability: social recognition memory formation. Our findings may offer additional information for understanding patients with social recognition deficits and finding appropriate treatment since electrolytic lesions resembles clinical lesion conditions^29^.

## Methods

### Animals

C57BL/6N (Takonic) male mice (8–12 weeks old, n = 24) were used in all experiments. Age-matched adult C57BL/6N male mice (n = 32) were used as social stimuli in this study. All mice were housed in a controlled environment (temperature; humidity; 12-h light/dark cycle) and given ad libitum access to food and water. All procedures were approved and conducted in accordance with the guidelines and regulations of the Ewha Womans University Institutional Animal Care and Use Committee (EWHA IACUC 21–008-t). Our research also follows the ARRIVE 2.0 guidelines^30^.

### Surgery

To investigate the role of aIC in social behaviors, behaviors of three groups (aIC lesion, sham, control) were compared. Lesion group received bilateral electrolytic aIC lesions, sham group underwent the same surgical procedures as the lesion group without current passing through the lesion electrodes, and control group underwent identical handling and housing procedures as the other groups without surgeries. A week before surgery, all groups of mice were housed with a cage mate. A transparent plastic divider with holes was placed in each cage, so mice were able to sniff each other without any physical interactions. A week before the experiment, surgical procedures were performed in the lesion and sham groups. The electrolytic lesion method was used to investigate the influence of a permanent lesion of the aIC in processing social abilities, since permanent lesion methods are suggested to mimic clinical conditions following chronic lesions^31^. On the day of the surgery, mice for the lesion group were anesthetized using a low-flow integrated digital anesthetic vaporizer (Somnosuite, Kent Scientific) with isoflurane (SomnoSuite settings were 2.5% (350 mL/min) for induction and 1.5% (150 mL/min) for maintenance of anesthesia), hooked up to a stereotaxic apparatus, placed with a custom-made unipolar lesion electrode (26 gauge, Teflon insulated copper wire) bilaterally in the anterior insular cortex (AP, + 2.0 mm ; ML, ± 2.75 mm; DV, − 2.5 mm), and direct current (0.8 mA, 3 s) was passed through each hemisphere using a lesion-maker (LMD-53500, Ugo Basile, Italy)^32^. Sham groups underwent the same surgical procedures without current passing through the electrodes. Control groups did not have any surgery. Mice in all groups were handled for a week (5 min/day). All mice were housed in cages with a divider with their original cage mate for a week before the experiments.

### Three chamber sociality test

For sociability tests, a linear three chamber apparatus (45 × 10 × 21 cm) was used to minimize the exploration of unnecessary areas and to increase the number of visits to the targets^33^. Two removable chambers (target chambers; 10 × 10 × 20 cm) barred with 1-cm-spaced thin acrylic bars, to allow the subject mouse to interact with targets, were placed at both ends of the apparatus. Each target chamber contained a stranger mouse (C57BL/6N male, 8–12 weeks old; social stimulus), an inanimate object (non-social stimulus), or nothing (empty chamber). A nonmoving bobble-head doll (4 × 3 × 7 cm) was used as the inanimate object throughout the experiments. Placement of social or non-social stimuli was randomized for all experiments. The experiment consisted of four 10 min sessions (Fig. 2D). In the first session, subject mice were allowed to explore the apparatus freely with both target chambers empty (session 1: Habituation). In session 2 (sociability test), mice explored between a stranger I (social stimulus 1) and an inanimate object (non-social stimulus) placed in target chambers. In session 3 (social novelty recognition test), mice explored between the stranger I (social stimulus 1 introduced in session 2) and a novel stranger II (social stimulus 2). In session 4 (social novelty recognition test with cage mate), mice explored between a cage mate mouse (familiar) and a novel stranger III (social stimulus 3). Cage mate mice were mice that were housed together before aIC lesion was made and for at least 2 weeks before the start of the experiment^34, 35^. Each subject mouse was removed from the apparatus and placed in the home cage between successive sessions with inter session intervals of 2 min. The light intensity was controlled at ~ 35 lx since high lux illumination was reported to interfere with rodents’ social behaviors^36^.

### Data analysis of the three-chamber sociality test

Videotaped behaviors were manually analyzed by at least two investigators (mean values used). The duration of investigation with a target were quantified as measures of interaction. The onset of a target investigation was defined as when a subject mouse’s nose entered the target chamber bars (manually analyzed). Investigations that lasted longer than 1 s with an interval greater than 2 s from the previous investigation were counted.

To compare the behavioral differences between the three groups (control, sham, and aIC lesion), direction preference index, sociability index, social novelty recognition index, and social novelty recognition index with cage mate were calculated using the following formulas.

$$Direction\,preference\,index=\frac{(\mathrm{Interaction\,time\,with\,left\,chamber}-\mathrm{Interaction\,time\,with\,the\,right\,chamber})}{(\mathrm{Interaction\,time\,with\,left\,chamber}+\mathrm{Interaction\,time\,with\,the\,right\,chamber})}$$

$$Sociability \,index=\frac{(\mathrm{Interaction\,time\,with\,conspecific }-\mathrm{Interaction\,time\,with\,non\,social\,object})}{(\mathrm{Interaction\,time\,with\,conspecific }+\mathrm{ Interaction\,time\,with\,non\,social\,object})}$$

$$Social\,novelty \,reocognition \,index=\frac{(\mathrm{Interaction\,time\,with\,novel\,stranger }-\mathrm{ Interaction\,time\,with\,familier}[\mathrm{earlier\,stranger}])}{(\mathrm{Interaction\,time\,with\,novel\,stranger\,}+\mathrm{\,Interaction\,time\,with\,familier}[\mathrm{earlier\,stranger}])}$$

$$Social\,novelty\,recognition\,index\,with\,cage\,mate=\frac{(\mathrm{Interaction\,time\,with\,novel\,stranger\,}-\mathrm{\,Interaction\,time\,with\,cage\,mate})}{(\mathrm{Interaction\,time\,with\,novel\,stranger }+\mathrm{\,Interaction\,time\,with\,cage\,mate})}$$

A one-way ANOVA followed by Tukey's multiple comparison test was used to compare the differences between the groups (see the statistical analysis section for more detail).

### Five-trial social novelty recognition test

To test whether aIC lesion affects the ability to recognize mice that had previously been encountered, five-trial social novelty recognition was carried out. Light intensity was controlled at ~ 35 lx throughout the experiment. In the experiment, a subject mouse was introduced into the center chamber and the test began when a stranger IV was introduced into the left target chamber and behaviors were recorded for 5 min. Stranger IV was introduced repeatedly for four trials with 10-min inter-trial intervals (ITIs). On the fifth trial, new novel mouse (stranger V) was introduced in the target chamber and behaviors were recorded. Videotaped locomotion during the tests was analyzed off line with Ethovision (Noldus, Netherlands). Investigation duration was scored manually using the same criterion as the three-chamber sociality test and familiarization index was calculated as follows.

$$Familiarization\,index=\frac{Interaction\,time\,with\,stranger\left(trial\,1\right)-Interaction\,time\,with\,stranger(trial\,4) }{Interaction\,time\,with\,stranger\left(trial\,1\right)+Interaction\,time\,with\,stranger(trial\,4)}$$

### Five-trial odorant novelty recognition test

A modified version of the conventional object recognition test was used to assess the non-social recognition memory of mice and determine whether the observed deficits in social novelty recognition were attributable to olfactory impairment^24, 37^. A non-social object (nonmoving bobble-head doll, 4 × 3 × 7 cm) and a cotton ball with 1 μl of methyl valerate (bubble gum scent, Sigma, 148,997) that was hidden behind non-social object were introduced to the left target chamber for the first four trials (Fig. 2E). In a fifth trial, the same non-social object and a cotton ball with 1 μl of α-Pinene (pineapple scent, Sigma, 147,524) were introduced. All trials were 5 min each with 10-min ITI. These two odorants were selected since male C57BL/6 mice could clearly distinguish them^38^. Light intensity was controlled at ~ 35 lx throughout the experiment. Videotaped locomotion during the tests was analyzed off line with Ethovision (Noldus, Netherlands). Investigation durations were scored manually using the same criteria previously described above and familiarization index was calculated as follows.

$$Familiarization\,index=\frac{Interaction\,time\,with\,object\left(trial\,1\right)-Interaction\,time\,with\,object(trial\,4) }{Interaction\,time\,with\,objtect\left(trial\,1\right)+Interaction\,time\,with\,object(trial 4)}$$

### Hidden food test

The hidden food test was used to examine possible deficits in the olfactory function of mice. Food was removed from the home cages, leaving only the water bottle, 24 h before the test. To habituate the environment for the hidden food test, mice were placed in a clean cage (46 × 23.5 × 20 cm) filled with a 3 cm depth of new bedding. After the habituation session, a 1.5 g food pellet was buried 1 cm beneath leveled bedding in a randomly chosen corner of the cage. Behavior was videotaped until the mice found the food pellet. The test was performed under low light intensity (~ 20 lx), and cage change was avoided throughout testing^39, 40^. Latency to find food was manually scored.

### Open field test

Open field test was used to examine the basic locomotor activity and general anxiety level of mice in the three groups. Mice were placed in the center of an open field box (50 × 50 × 50 cm opaque white acrylic) and allowed to explore the environment for 10 min. Using the Ethovision XT 16 (Noldus, Wageningen, Netherlands) software, total distance travelled, time spent in the center, and number of entries into the center were analyzed^41^. To analyze the behavior of the mice, 10 cm area around the walls of chamber was considered as peripheral, while the rest of the area was considered as the center^42^. During the test, indirect light (light intensity ~ 75 lx) was used^43^.

### Auditory fear conditioning and extinction

Fear conditioning experiment was carried out with a FreezeFrame system (Coulbourn Instruments, Allentown, PA). On the first day of fear conditioning all mice were habituated to the test room for 1 h before auditory fear conditioning. After habituation, a mouse was placed in a fear conditioning chamber (35 × 35 × 40 cm transparent chamber with stainless-steel grid floor for electrical shock delivery) and allow to explore for 3 min. Then, mice were fear conditioned with three pairs of conditioning stimulus (CS; tone: 80 dB, 10 kHz, 30 s) that co-terminated with an unconditioned stimulus (US; foot shock: 0.5 mA, 2 s). ITIs between CS-US pairing was 90 s. Mice were kept in the chamber for another 90 s after the last shock. The next day, the second day of the test, acquisition of cue (tone) fear conditioning was tested in a different chamber (white cylindrical chamber, 26 × 36 cm). Mice were allowed to explore for 3 min, then the same tone (80 dB, 10 kHz, 30 s) was played for three times with 180 s intervals. On the third day, to test contextual fear conditioning, mice were placed in the fear conditioning chamber and behaviors were recorded for 10 min. On the fourth and fifth day, fear extinction experiment was carried out in a white cylindrical chamber, used for the cued fear conditioning test. Mice were allowed to explore for 3 min, then they were repeatedly exposed to twenty CSs with variable inter-tone intervals 30-140 s per day^44^. Freezing was defined as the absence of all movement, aside from that required for respiration (without regard to posture), for a minimum of 0.75 s^45^. Time spent in freezing was scored manually by at least two investigators (mean values used).

### Histology

Locations of the electrolytic lesion were verified with histology. After intraperitoneal injection of 10% urethane (Sigma), mice were trans-cardially perfused with saline (0.9%) followed by formalin (10% formalin diluted in saline). Brains were removed and stored in formalin (10% formalin diluted with deionized water) for a day and transferred to a 30% sucrose solution for another day for further fixation. Fixed brain tissues were cut in coronal Sects. (40 µm-thick) through the anterior insular cortex with a vibratome (Precisionary Instruments LLC). Brain slices were mounted on slides, allowed to dry for a day, and stained with a cresyl-violet solution (Sigma, USA) for 10 min. Lesion sites were examined with a light microscope (Zeiss, Axioscope 5). Only mice with more than 40% of the aIC area lesioned in both hemispheres were included in the analysis of the lesion group. Size of lesions (% of aIC area) were analyzed with ImageJ (NIH, USA)^46^. Mean percentages of the left and right aIC lesions are shown in Supplementary Fig. S1.

### Statistical analyses

Statistical analyses were performed with the SPSS 26.0 (SPSS Inc., USA). Normality of data was analyzed with the Shapiro–Wilk normality test. Data were determined to satisfy normal distributions and, therefore, a paired t-test was used to assess differences of within group preferences in the linear chamber test. One-way analysis of variance (ANOVA) with post hoc Tukey’s test was used to compare differences between the three groups (lesion, sham, control) in the linear chamber test, open field test, hidden food test, baseline freezing level, cued and contextual fear, and movement speed for all behavior tests. A two-way repeated measures ANOVA with Bonferroni post-hoc test was used to analyze changes over trials 1–4 of the five-trial tests, fear memory acquisition, and fear extinction learning.

## Supplementary Information

Supplementary Information.

## Data Availability

The datasets used in the current study will be available from the corresponding author on reasonable request.
