# Supplementary material for: The anterior insular cortex processes social recognition memory
Source: Sci Rep. 2023 Jul 5;13:10853. doi: 10.1038/s41598-023-38044-6 (PMC10322941; doi:10.1038/s41598-023-38044-6)
Supplement: Supplementary file 1 — Supplementary Information. [file 41598_2023_38044_MOESM1_ESM.pdf]

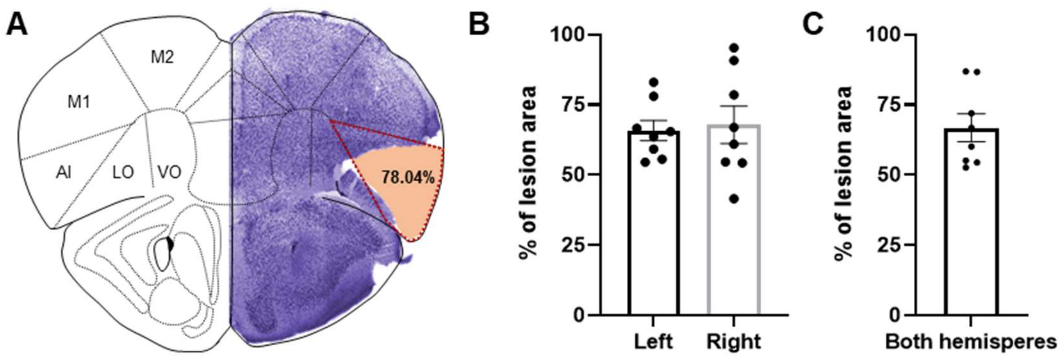

Supplementary Fig. S1. Mean percentages of complete aIC lesions in the left and right hemispheres

(A) Representative pictures of cresyl-violet stained brain slices from the aIC-lesion group. The area outlined by the red dotted line indicates the aIC region. The area shaded with apricot color indicates the region of complete ablation.

(B) Mean percentages of aIC lesion area in the left and right hemispheres.

(C) Mean percentages aIC lesion area of both hemispheres.

(B and C) All data are presented as mean ± SEM.
